# The effect of L-theanine supplementation on the immune system of athletes exposed to strenuous physical exercise

**DOI:** 10.1186/s12970-019-0274-y

**Published:** 2019-02-15

**Authors:** A. Juszkiewicz, A. Glapa, P. Basta, E. Petriczko, K. Żołnowski, B. Machaliński, J. Trzeciak, K. Łuczkowska, A. Skarpańska-Stejnborn

**Affiliations:** 1 Department of Morphological and Health Sciences, Faculty of Physical Culture in Gorzów Wielkopolski, 13 Estkowskiego Str, 66-400 Gorzów Wielkopolski, Poland; 2 Department of Water Sports, Faculty of Physical Culture in Gorzów Wielkopolski, 13 Estkowskiego Str, 66-400 Gorzów Wielkopolski, Poland; 3 Department of Pediatrics, Endocrinology, Diabetology, Metabolic Disorders and Cardiology of Developmental Age, Pomeranian Medical University, Unii Lubelskiej Str, 71-252 Szczecin, Poland; 4 Department of General Pathology, Pomeranian Medical University, 72 Powstanców Wielkopolskich Alley, 70-111 Szczecin, Poland; 5 Faculty of Physical Culture in Gorzów Wielkopolski, 13 Estkowskiego Str, 66-400 Gorzów Wielkopolski, Poland

**Keywords:** L-theanine, Inflammation, Flow cytometry, Cytokine, Strenuous exercise, Rowers

## Abstract

**Background:**

The aim of this study was to analyze the response of selected components of the immune system in rowers to maximal physical exercise, and to verify if this response could be modulated by supplementation with L-theanine*.*

**Method:**

The double-blind study included 20 members of the Polish Rowing Team. The subjects were randomly assigned to the supplemented group (*n* = 10), receiving 150 mg of L-theanine extract for 6 weeks, or to the placebo group (*n* = 10). The participants performed a 2000-m test on a rowing ergometer at the beginning (1st examination) and at the end of the supplementation period (2nd examination). Blood samples were obtained from the antecubital vein before each exercise test, 1 min after completing the test, and after a 24-h recovery. Subpopulations of T regulatory lymphocytes (Tregs) (CD4+/CD25+/CD127-), cytotoxic lymphocytes (CTLs) (CD8+/TCRαβ+), natural killer (NK) cells (CD3-/CD16+/CD56+) and TCRδγ-positive (Tδγ) cells were determined by means of flow cytometry. The levels of interleukin 2 (IL-2), interleukin 4 (IL-4), interleukin 10 (IL-10), interferon gamma (INF-ɤ) and total antioxidant capacity (TAC) were determined with commercially available diagnostic kits.

**Results:**

Supplementation with L-theanine contributed to a significant post-exercise decrease in IL-10 concentration, which was reflected by higher values of IL-2 to IL-10 and IFN-γ to IL-10 ratios. Moreover, a significant post-recovery decrease in CTL count, Treg to NK and Treg to CTL ratios was observed in the supplemented group.

**Conclusion:**

Despite the decrease in the number of some cytotoxic cells (CTLs) and an increase in the proportion of Tregs to CTLs, supplementation with LTE seems to exert a beneficial effect on a disrupted Th1/Th2 balance in elite athletes, as shown by the decrease in IL-10 concentration.

## Introduction

Elite athletes, exposed to extremely large training loads, may develop the so-called overtraining syndrome (OTS) which may inter alia contribute to a decrease in their performance [1, 2]. One consequence of OTS is greater susceptibility to upper respiratory tract infections (URTI), which suggests that overtraining is associated with immune impairment [3]. Recent evidence showed that the immune impairment in OTS may be caused by a disruption of Th1/Th2 balance. Human T-helper (Th) lymphocytes are represented by two distinct functional subsets: Th1 cells, causing activation of cell-mediated immunity associated with the elimination of intracellular pathogens and anticancer protection, and Th2 cells, stimulating humoral immunity associated with antibody synthesis. Previous studies demonstrated that strenuous exercise may contribute to cellular immunosuppression and resultant shift in Th1/Th2 balance toward Th2, which results in greater susceptibility to infection, inflammation and overtraining [4–6]. This justifies the inclusion of supplements with established immunomodulatory effects in athletes’ diet, as an element of a mild and safe intervention aimed at restoration of immune balance.

L-theanine (LTE) is a main amino acid present in tea leaves. LTE is metabolized in kidneys to glutamic acid and ethylamine [7]. The latter is an alkylamine, and aside from the tea leaves, can also be found (albeit at a lower amount) in other products, e.g. mushrooms, apples, red wine, to mention a few [8]. Previous studies showed that LTE may modulate immunity, regulating secretion of Th1 and Th2 cytokines. Animals supplemented with LTE presented with higher serum concentrations of interleukin-2 (IL-2) and interferon gamma (IFN-γ) [9]. Another study showed that administration of LTE conditioned Tγδ lymphocytes for stronger response to the stimulus triggering cytotoxic reaction. Drinking black tea contributed to a significant increase in the secretion of IFN-γ by Tγδ lymphocytes from healthy volunteers, after in vitro stimulation with bacterial antigens [8]. In turn, addition of ethylamine to the culture of peripheral blood mononuclear cells obtained from healthy volunteers resulted a 15-fold increase in Tγδ count [10]. At low, micromolar, concentrations alkylamines maintain Tγδ readiness for a rapid response to invasion of pathogenic or opportunistic bacteria; the number of Tγδ cells and the synthesis of antibacterial cytokines (among them IFN-γ) were shown to increase substantially following exposure to bacterial antigens. However, without antigenic stimulation, markedly higher, millimolar, concentrations of alkylamines are required to induce the proliferation of Tγδ cells [8].

Murakami et al. [11] analyzed the effects of supplementation with LTE and cystine on some immune functions in athletes after a long run. While a decrease in lymphocyte count was documented after the run in non-supplemented controls, no similar effect was observed in the supplemented group. The same study showed a statistically significant increase in the activity of natural killer (NK) cells; although this effect was observed in both groups, it was markedly more evident in the athletes supplemented with LTE and cystine. According to Kawada et al. [12], the post-exercise activation of NK cells in well-trained men supplemented with LTE and cystine was significantly stronger than in non-supplemented controls. In both studies mentioned above, LTE was administered together with cystine; this regimen was chosen based on the results of a previous animal study in which mice received cystine and/or LTE. The study showed that stimulation with theanine and cystine contributed to a substantial increase in the synthesis of IgG following antigenic stimulation. Moreover, animals supplemented with LTE and cystine presented with significantly higher values of IL-10 to IFN-γ ratio. In contrast, the values of this parameter in mice that received LTE alone were lower than at the baseline, which probably resulted from a significant decrease in IL-10 level, observed already within 24 h of the supplementation [13].

As shown above, previous studies demonstrated that both cystine and intense physical exercise may promote a shift in Th1/Th2 balance toward Th2. Thus, we hypothesized that supplementation with LTE may promote Th1 response and attenuate Th2 response in elite athletes. One method for functional assessment of the Th1/Th2 balance is determination of Th1 (e.g. INF-γ, IL-2) and Th2 cytokine (e.g. IL-4, IL-10) levels. However, to accurately estimate the status of Th1/Th2 balance, the levels of cytokines produced by Th1 and Th2 lymphocytes should be interpreted together, since an increase in Th1 cytokine concentration may be frequently counterbalanced by an increase/decrease in Th2 cytokine level, and vice versa [14, 15]. Therefore, the analysis of a shift in Th1/Th2 balance triggered by strenuous physical exercise (an extreme stressor) and supplementation (LTE) based on the values of INF-γ to IL-4, INF-γ to IL-10, IL-2 to IL-4 and IL-2 to IL-10 ratios would be more accurate than merely the analysis of changes in concentrations of individual cytokines. Another parameter to be considered during the evaluation of immune balance after strenuous physical exercise is the number of cytotoxic cells (e.g. NK, CLT, Tγδ) and T-regulatory lymphocytes (Tregs).

Since other authors demonstrated that various lymphocyte fractions can interact with one another, either directly or via synthesized cytokines, we searched for sufficiently accurate markers of those relationships. Based on the observations published by other researchers [16–19] who demonstrated that Treg to effector T-cell ratios (Treg to Tγδ, Treg to NK, Treg to CTL, Treg to NK + TCR + CLT) are more accurate measures of the suppressive effects exerted by the regulatory lymphocytes than a simple cytometry, we also considered those indices in our study. To the best of our knowledge, this study was the first one to use such comprehensive approach to the analysis of exercise-induced changes in Th1/Th2 balance in elite athletes.

## Materials and methods

### Study population

The study included 20 men, all members of the Polish Rowing Team (16 heavy-weight and 4 light-weight rowers). Basic characteristics and sport classes of the athletes are presented in Table 1. The study was conducted between March and May, during a 6-week training camp scheduled between the preparatory and competitive phase of the yearly training cycle. The characteristics of the training profile, such as its intensity, volume (in minutes) and type (specific, i.e. rowing: endurance, technical, speed, etc., and nonspecific: jogging, strength) were recorded on a daily basis. The intensity of the training was classified based on the lactate acid (LA) threshold (4 mmol/L), as extensive (below the LA threshold), highly intensive (above the LA threshold), and extremely intensive (control tests) (Table 2).

**Table 1:** Basic characteristics of the study groups

Parameters	Supplemented group (*n* = 10)	Placebo group (*n* = 10)
Age (years)	21.0 ± 0.91	20.5 ± 1.08
Body weight (kg)	88.9 ± 7.02	85.7 ± 10.40
Body height (cm)	191.2 ± 3.03	187.9 ± 5.62
Duration of training (years)	7.5 ± 1.5	6.3 ± 1.5

Values represent means ± standard deviations. No statistically significant differences were found for all intergroup comparisons (*P* > 0.05)

**Table 2:** Training schedule for the week preceding blood sampling during the 1st and the 2nd examination

	Days before the 1st examination						
	1	2	3	4	5	6	7
Total training time, min/day	120	110	200	120	220	110	90
Time rowed, min/day	105	–	100	110	100	100	–
Distance rowed, km/day	18	–	20	22	20	20	–
Training for force development, min/day	–	–	90	–	90	–	80
Extensive endurance rowing training time, min/day	81	–	100	65	70	100	–
High intensity endurance rowing training time, min/day	24	–	–	45	30	–	–
Unspecific training (running, etc.), min/day	15	110	10	10	30	10	10
	Days before the 2nd examination						
	1	2	3	4	5	6	7
Total training time, min/day	190	90	190	110	200	100	80
Time rowed, min/day	100	80	100	100	100	90	–
Distance rowed, km/day	20	16	18	18	20	18	–
Training for force development, min/day	–	–	80	–	90	–	–
Extensive endurance rowing training time, min/day	84	80	100	52	67	90	–
High intensity endurance rowing training time, min/day	-	-	-	48	12	20	5
Very high intensity endurance rowing training time, min/day	16	-	-	-	21	-	-
Unspecific training (running, etc.), min/day	90	10	10	10	20	10	80

### Food intake

Throughout the study period, the athletes were accommodated at an Olympic Training Center, whereby they had all their meals. Their regular menu consisted of a mixed diet, providing the recommended dietary allowance (RDA) of carbohydrates, proteins, fats and micronutrients (vitamins and minerals), in line with the Polish Nutrition Society guidelines [20]. Daily intakes of food, calories, fruits and vegetables were the same throughout the study period. None of the athletes drank more than two cups of tea per day. Average L-theanine content per single cup of tea approximates 7.9–24.2 mg [21].

The study subjects declared that they had ceased all drugs, medications and dietary supplements at least 2 weeks before the experiment, and did not use them throughout the entire study period.

### Experimental procedure

Athletes who were randomized to the supplemented group (*n* = 10) received gelatin capsules with 150 mg L-theanine extract, manufactured by Nanga (Złotów, Poland)*.* The subjects were asked to take two capsules per day for 6 weeks. The dose was selected based on published data [22, 23]. Athletes randomized to the placebo group (*n* = 10) received visually identical capsules with cornstarch (150 mg per capsule)*.*

### Training program

Training volumes (expressed in minutes per day) during a week preceding the 1st and the 2nd examination are shown in Table 2, separately for extensive rowing, intensive rowing, kilometers and extensive non-specific training. During the load training phase (before the 1st examination), the training volume amounted to 970 min per week, including approximately 42.9% of extensive rowing, 10.2% of non-specific training (e.g. power training) and 20.1% of intensive rowing. Total training volume before the 2nd examination was 960 min·per week and included approximately 48.2% of extensive rowing, 7.3% of intensive rowing (with 3.9% of maximum-intensity control tests).

### Rowing performance test

The athletes performed a controlled 2000-m time trial on the first day (before the supplementation) and at the end of the training camp (after the supplementation). Each subject had to cover the 2000-m distance on a rowing ergometer (Concept II, USA) in the shortest time possible. Because the results of both tests were taken into consideration during selection to the championship team, the athletes were motivated well to perform both tests at a maximal effort. Before each test, the subjects performed a 5-min individual warm-up on the ergometer.

### Sample treatment

Blood samples from the antecubital vein were collected to tubes with dipotassium ethylene diamine tetra-acetic acid (K_2_EDTA) as an anticoagulant. Blood was collected before each 2000-m test (after 7- to 8-h overnight fast), 1 min after completing the test, and after a 24-h recovery. The list of analyzed parameters included white blood cell (WBC) counts and percentages of lymphocytes and granulocytes, all determined with MYTHIC 18 Hematology Analyzer (Orphee Medical, Geneva, Switzerland). The samples collected to tubes with the anticoagulant were centrifuged for 10 min at 2200 rpm. After removing the plasma and adding 1x Lysing Buffer (BD Biosciences), the samples were incubated in darkness for 15 min. Then, PBS buffer was added, and the cells were washed twice to remove all erythrocytes. Blood samples collected to the tubes without additives were centrifuged for 10 min at 3500 rpm, and the sera were stored at − 80 °C until the analysis. Moreover, capillary blood samples from the earlobe were collected before and after each exercise test, to assess LA level.

### Measurements

Cytometric analysis of lymphocyte subpopulations: Tregs (CD4+/CD25+/CD127-), CTLs (CD8+/TCRαβ+), NK cells (CD3-/CD16+/CD56+) and Tδγ cells was conducted after their labeling with fluorochrome-conjugated antibodies from BD Biosciences. The cells obtained after hypotonic lysis of peripheral blood were incubated in darkness at room temperature for 20 min with the respective antibody at a concentration specified by the manufacturer, to identify each lymphocyte subpopulation (Table 3). Then, the cells were washed twice with PBS buffer and left in darkness in 3.7% formaldehyde solution for 10 min. Subsequently, the cells were again washed with PBS buffer, and 100 μl of DAPI solution (1 mg/ml, Thermo Fisher Scientific) were added to stain cell nuclei. Then, the cells were incubated in darkness at room temperature for 5 min, washed twice, and suspended in 250 μL of PBS buffer. After labeling, the cells were analyzed with LSRII flow cytometer (BD Biosciences) coupled with BD FACSDiva software.

**Table 3:** Antibodies used for identification of lymphocyte subpopulations

Lymphocyte subpopulation	Antibody
Regulatory T lymphocytes	FITC Mouse Anti-Human CD4 PE Mouse Anti-Human CD25 Alexa Fluor 647 Mouse anti-Human CD127
Cytotoxic lymphocytes	APC Mouse Anti-Human CD8 FITC Mouse Anti-Human TCR αβ
NK cells	FITC Mouse Anti-Human CD3 PE Mouse Anti-Human CD16 APC Mouse Anti-Human CD56
TCRδ/γ lymphocytes	FITC Mouse Anti-Human TCRδ/γ

Serum concentrations of interleukins: IL-2, IL-4 and IL-10 were measured using a commercially available enzyme immunoassays (ELISA; Abcam, Cambridge, UK) with detection ranges of 1.87–60 pg/ml, 0.31–10 pg/ml and 1.56–50 pg/ml, respectively. Serum levels of interferon gamma (IFN-γ) were quantified using a commercially available enzyme immunoassay (ELISA; Quantikine HS, R&D Systems, Minneapolis, USA) with a detection range of 15.6–1000 pg/ml.

Total antioxidant capacity (TAC), considered a marker of plasma antioxidant capacity, was assessed with a commercially available kit (LDN Labor Diagnostika Nordhorn, Germany) with an assay range of 0.375–3 mmol/l. Concentration of LA in capillary blood was determined immediately after sampling, using a commercially available kit (Dr Lange, Germany); LA concentrations were expressed in mmol/l. The coefficients of variation for all assays were < 11%.

### Statistical analysis

Statistical analyses were conducted with STATISTICA v. 13.1 software package (StatSoft, Cracow, Poland). All parameters were compared using 2 (supplementation: supplemented vs. placebo) × 3 (exercise: pre-exercise vs. post-exercise vs. post-recovery) repeated measures analysis of variance (ANOVA). Normal distribution of the study variables was verified with Shapiro-Wilk test. Whenever the result of ANOVA was statistically significant, Fisher’s *post-hoc* test was conducted to identify the source of significant differences. Anthropometric characteristics of the study groups were compared with unpaired Student’s *t*-test. Except the rowing time, the results of the 2000-m tests performed before and after the supplementation were compared with paired Student’s *t*-test, and intergroup comparisons were conducted with unpaired Student’s *t*-test. The results of the 2000-m simulated rowing test were subjected to one-way ANOVA. Statistical characteristics of the study variables are presented as means ± standard deviations (SD), and the threshold of statistical significance for all tests was set at *p* < 0.05.

## Results

Athletes from the supplemented group and the placebo group did not differ significantly in terms of their mean age, body height, body weight and years of training (Table 1).

No significant intragroup differences were found in mean power output and total run time during the 2000-m test performed at the beginning and at the end of the training camp (1st and 2nd examination, respectively). Furthermore, no significant intragroup differences in the pre- and post-supplementation blood LA levels were documented (Table 4).

**Table 4:** Changes in 2000 m rowing ergometer performance before and after supplementation with LTE

Parameters	Supplemented group (*n* = 10)		Placebo group (*n* = 10)	
	Before	After	Before	After
Power (Watt)	436 ± 28.5	445 ± 17.52	401 ± 31.48	411 ± 36.69
(W x kg^−1^)	5.01 ± 0.37	5.02 ± 0.28	4.81 ± 0.44	4.93 ± 0.22
LA_min_ (mmol x L^−1^)^a^	1.6 ± 0.37	1.5 ± 0.81	1.8 ± 0.73	1.6 ± 0.25
LA_max_ (mmol x L^−1^)^a^	15.1 ± 2.31	11.6 ± 2.42	14.1 ± 3.18	12.3 ± 4.13
Time (s)	371.3 ± 8.47	368.7 ± 5.22	382.1 ± 12.98	379.2 ± 11.78

Values represent means ± standard deviations. ^a^LA, lactate. No statistically significant differences were found between the pre- and post-supplementation results (*P* < 0.05)

**Fig. 1:**
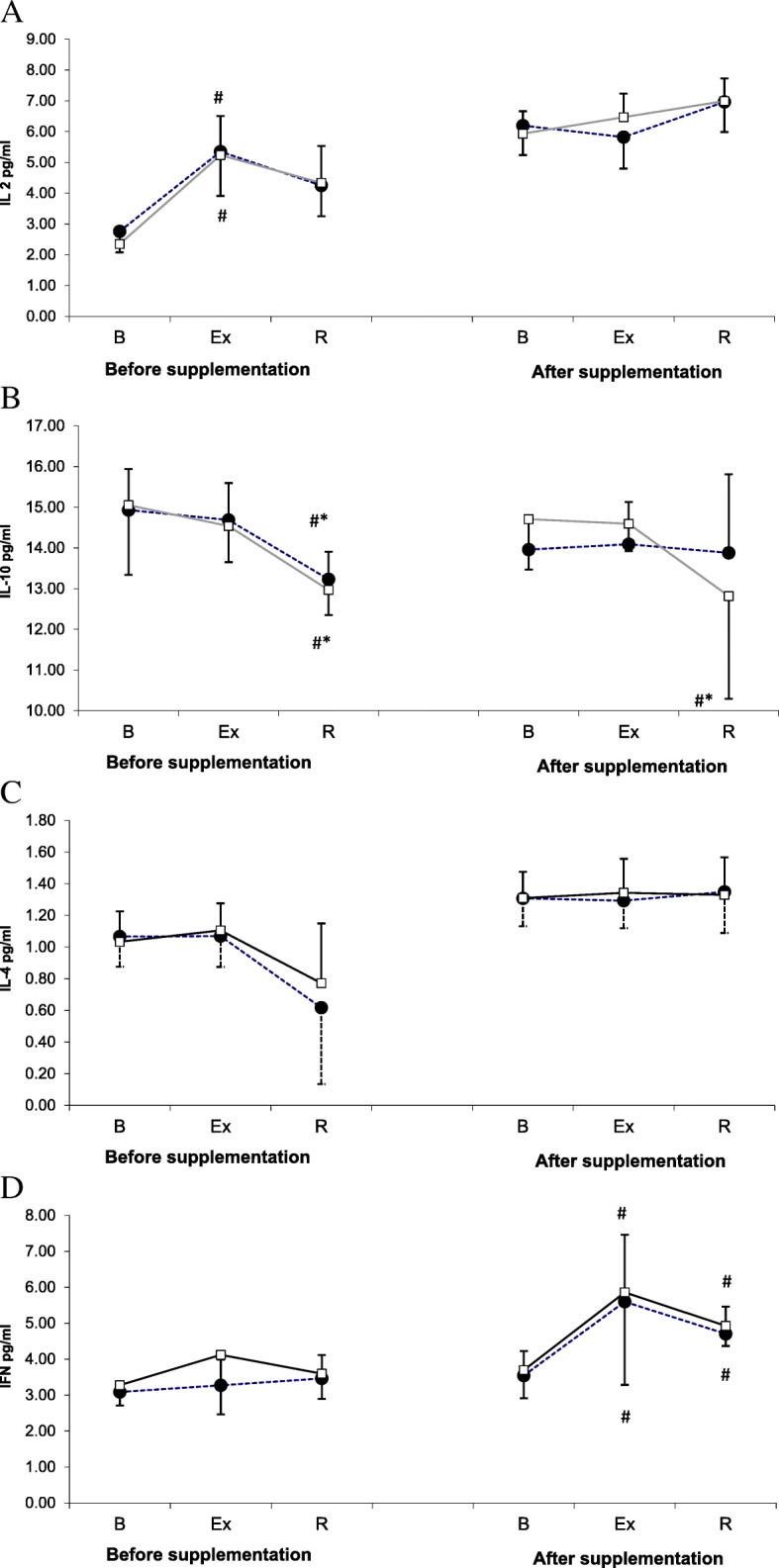
Changes in IL-2 (**a**), IL-10 (**b**), IL-4 (**c**), and IFN-gamma (**d**) levels during exercise tests performed prior to and after supplementation with LTE (mean ± *SD*). *Note*. IL = interleukins; INF γ = interferon gamma**; □** − SUPPL = supplemented group; ● − PLA = placebo group; B = baseline; Ex = post-exercise; R = after a 1-day recovery; # - significantly different compared to the baseline level; * - significantly different compared to the post-exercise level

During the 1st examination, a significant post-exercise increase in IL-2 level was observed in both study groups (Fig. 1a). However, no statistically significant exercise-induced changes in the concentration of this cytokine were documented during the 2nd examination. Furthermore, this parameter turned out to be independent of L-theanine supplementation (ANOVA, main effect, *p* = 0.981).

Changes in IL-10 levels in the study athletes are presented in Fig. 1b. During the 1st examination, the post-recovery levels of IL-10 in both study groups were significantly lower than at the baseline and immediately after the exercise test. Although supplementation with L-theanine did not exert a significant effect on IL-10 level (main effect, *p* = 0.943), during the 2nd examination, the post-recovery concentration of this cytokine in the supplemented group was significantly lower than at the baseline and immediately after the exercise.

The levels of IL-4 at various study timepoints are presented in Fig. 1c. This parameter turned out to be modulated by neither physical exercise (main effect, *p =* 0.662) nor supplementation (main effect, *p =* 0.508).

No statistically significant changes in IFN-γ levels were observed during the 1st examination (Fig. 1d). In contrast, a post-exercise increase in this parameter, which also persisted after a 24-h recovery, was demonstrated in both groups during the 2nd examination. Statistical analysis showed that supplementation with L-theanine did not exert a significant effect on IFN-γ levels (main effect, *p =* 0.172).

**Fig. 2:**
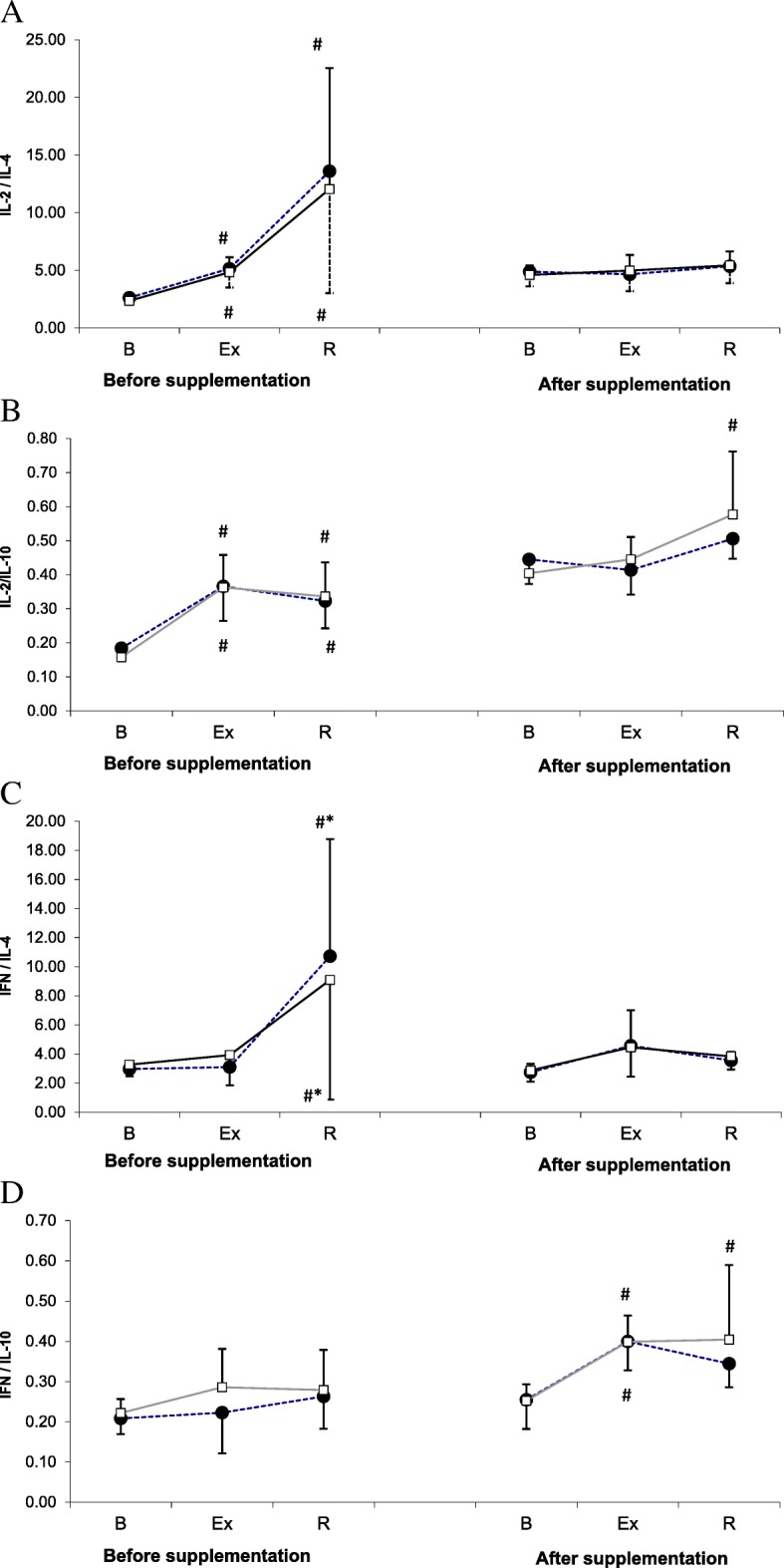
IL-2/IL-4 (**a**), IL-2/IL-10 (**b**), IFN-γ/IL-4 (**c**) and IFN-γ/IL-10 (**d**) ratios during exercise tests performed before and after supplementation with LTE (mean ± *SD*). *Note*. IL = interleukins; INF γ = interferon gamma**; □** - SUPPL = supplemented group; ● − PLA = placebo group; B = baseline; Ex = post-exercise; R = after a 1-day recovery; # - significantly different compared to the baseline level; * - significantly different compared to the post-exercise level

The values of proinflammatory to anti-inflammatory cytokine ratios are presented in Fig. 2. Supplementation with L-theanine did not influence significantly any of the analyzed indices of Th1/Th2 balance. However, ANOVA demonstrated that physical exercise exerted significant effects on the values of all ratios (main effect *p* < 0.001 for all analyzed parameters).

During the 1st examination, physical exercise contributed to a significant increase in the values of IL-2 to IL-4 and IL-2 to IL-10 ratios in both groups, and this effect also persisted after a 24-h recovery (Fig. 2a and b). During the 2nd examination, the post-recovery values of IL-2 to IL-4 ratio in the supplemented group, but not in the placebo group, were significantly higher than at the baseline.

Statistically significant changes in IFN-γ to IL-4 ratio were demonstrated solely during the 1st examination when the post-recovery values of this parameter in both study groups were significantly higher than at the baseline and immediately after the exercise (Fig. 2c). In turn, statistically significant changes in IFN-γ to IL-10 ratio were observed solely during the 2nd examination, when athletes from both groups showed a significant post-exercise increase in this parameter. After a 24-h recovery, the values of IFN-γ to IL-10 ratio in the supplemented group, but not in the placebo group, were still significantly higher than at the baseline (Fig. 2d).

**Fig. 3:**
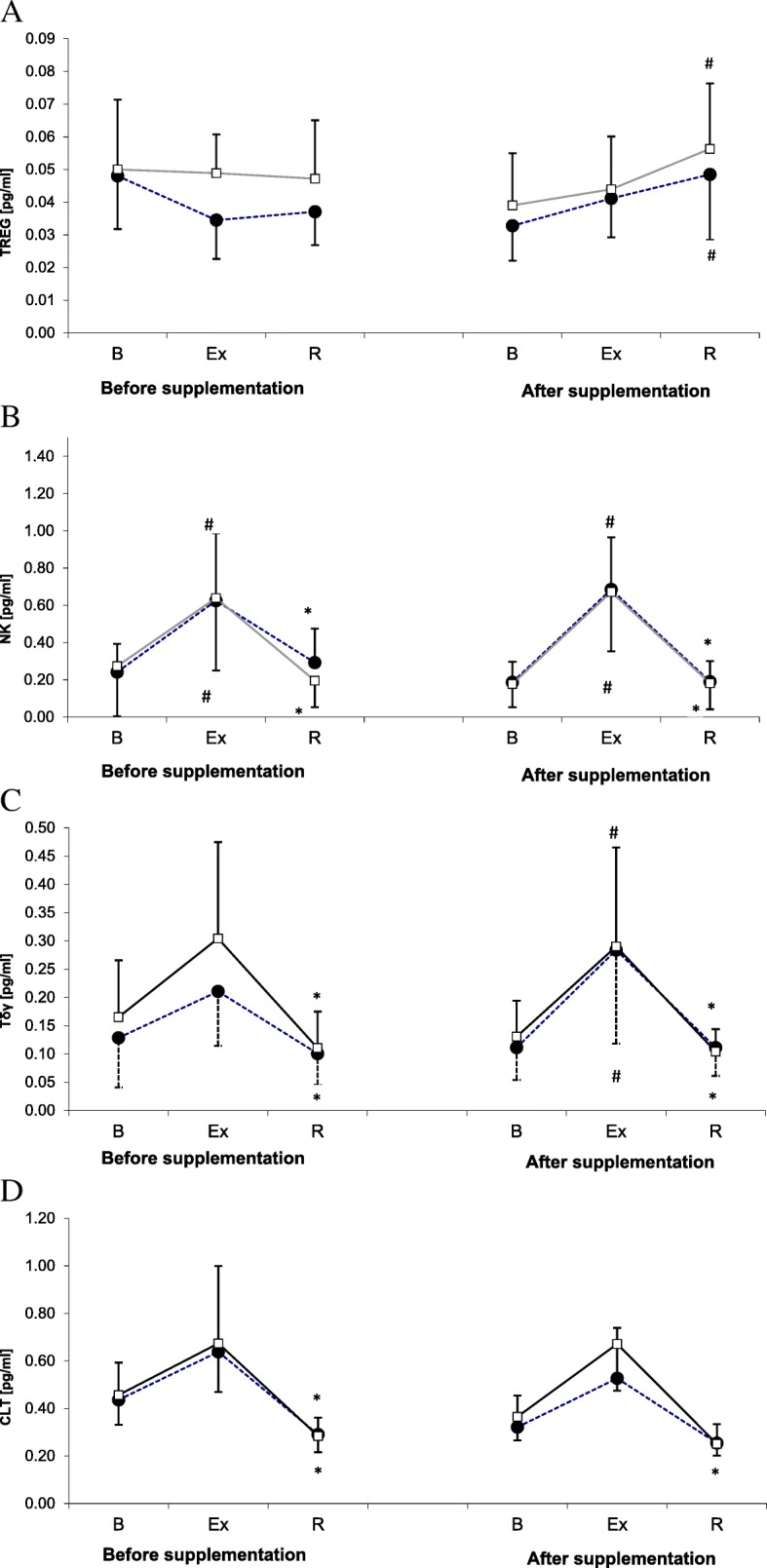
Changes in Treg (**a**), NK cell (**b**), Tδγ cell (**c**) and CTL (**d**) counts during exercise tests performed prior to and after supplementation with LTE (mean ± *SD*). *Note*. Tregs = regulatory T cells; NK = natural killer cells; Tδγ *= gamma delta T cells*; CLT = cytotoxic T lymphocytes; **□** − SUPPL = supplemented group; ● − PLA = placebo group; B = baseline; Ex = post-exercise; R = after a 1-day recovery; # - significantly different compared to the baseline level; * - significantly different compared to the post-exercise level

Treg counts determined during the 1st and the 2nd examination are shown in Fig. 3a. ANOVA revealed a significant main effect of exercise on Treg count (*p* < 0.001). During the 2nd examination, the post-recovery Treg counts in athletes from both groups were significantly higher than at the baseline (*p <* 0.05). However, ANOVA demonstrated that supplementation with L-theanine did not exert a statistically significant effect on Treg count (main effect, *p* = 0.149).

NK cell counts determined before and after supplementation with L-theanine are shown in Fig. 3b. ANOVA demonstrated a significant main effect of exercise on NK cell count (*p* < 0.001). During both the 1st and the 2nd examination, a significant post-exercise increase in this parameter was observed in both supplemented group and placebo group, followed by a post-recovery normalization at the baseline level.

Tδγ cell counts in the study subjects are presented in Fig. 3c. Likewise for other lymphocyte subpopulations, ANOVA demonstrated a significant main effect of exercise on Tδγ cell count (*p* < 0.001). During the 1st examination, the post-recovery Tδγ cell counts in both groups were significantly lower than immediately after the exercise. During the 2nd examination, physical exercise contributed to a significant increase in Tδγ cell counts in both groups, with subsequent return of this parameter to its baseline levels following a 24-h recovery. The main effect of the supplementation on Tδγ cell count did not turn out to be statistically significant (*p* = 0.156).

ANOVA demonstrated that exercise exerted a significant main effect on CTL count (*p* < 0.001). Irrespective of the study group, a significant post-recovery decrease in CTL count was observed during both the 1st examination (Fig. 3d). During the 2nd examination, however, the post-recovery decrease was documented solely in the supplemented group. Nevertheless, the main effect of the supplementation on CTL count did not turn out to be statistically significant on ANOVA (*p* = 0.294).

**Fig. 4:**
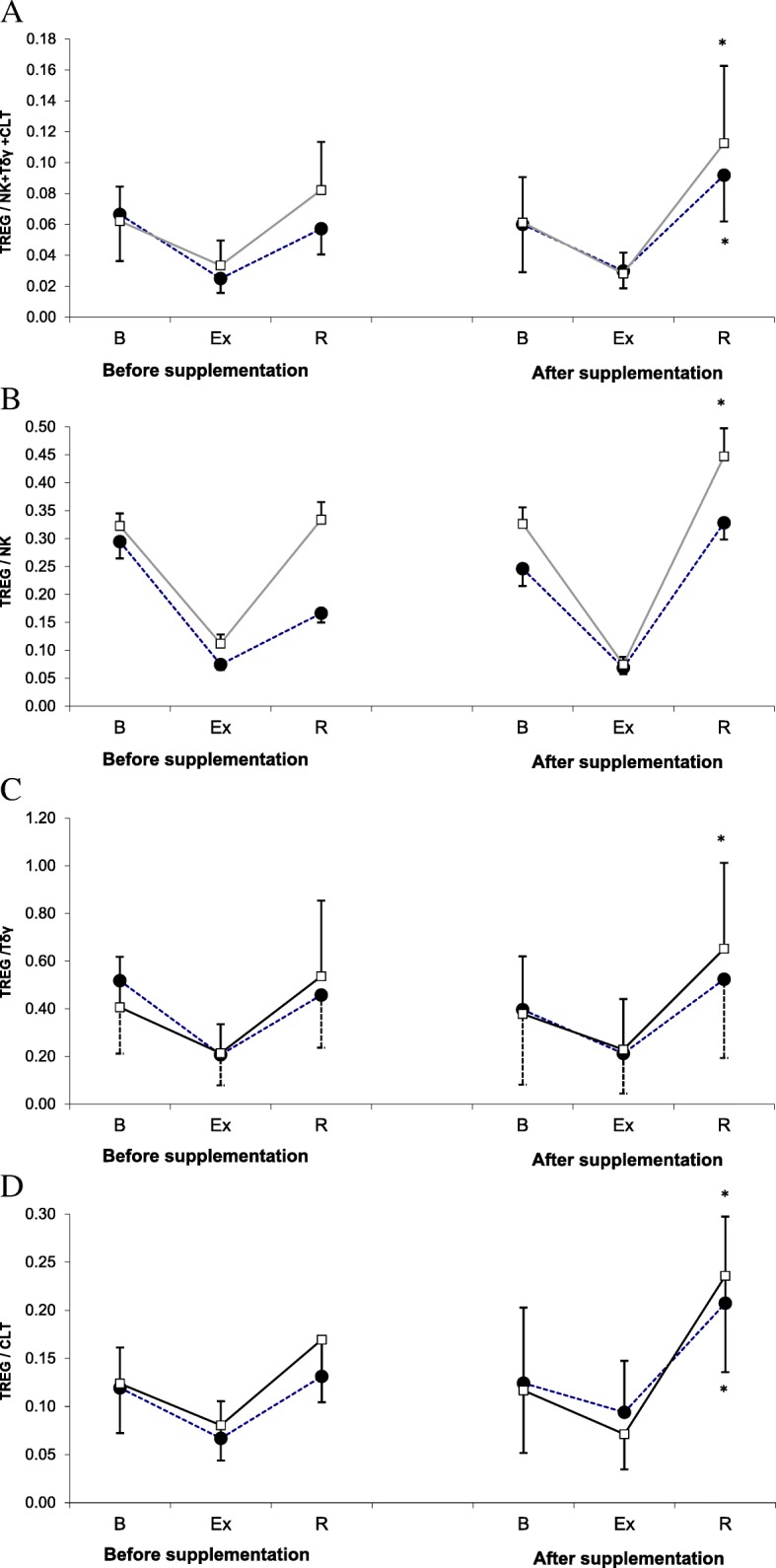
Treg/(NK + Tδγ + CTL) ratio (**a**), Treg/NK ratio (**b**), Treg/Tδγ ratio (**c**) and Treg/CTL (**d**) ratio during exercise tests performed prior to and after supplementation with LTE (mean ± *SD*). *Note*. Tregs = regulatory T cells; NK = natural killer cells; Tδγ *= gamma delta T cells*; CTL = cytotoxic T lymphocytes; **□** − SUPPL = supplemented group; ● − PLA = placebo group; B = baseline; Ex = post-exercise; R = after a 1-day recovery; # - significantly different compared to baseline level; * - significantly different compared to post-exercise level

The values of Treg to (NK + Tδγ + CTL) ratio are presented in Fig. 4a. No statistically significant changes in this parameter were documented during the 1st examination. During the 2nd examination, the post-recovery values of Treg to (NK+ Tδγ + CTL) ratio in both supplemented group and placebo group turned out to be significantly higher than at the baseline. In line with these finding, ANOVA showed that the main effect of the supplementation on Treg to (NK+ Tδγ + CTL) ratio was not statistically significant (*p* = 0.053).

Statistical analysis showed that the values of Treg to NK ratio were influenced by physical exercise (*p* < 0.001), but not by the supplementation (*p =* 0.233). Irrespective of the examination term, the post-exercise values of Treg to NK ratio in both study groups were lower than at the baseline, but this effect was not statistically significant (Fig. 4b). However, during the 2nd examination, the post-recovery Treg to NK ratio in the supplemented group turned out to be significantly higher than immediately after the exercise.

Exercise-induced changes in Treg to Tδγ ratio values are presented in Fig. 4c. This parameter turned out to be significantly modulated by strenuous physical exercise (main effect, *p* < 0.001), but not by the LTE supplementation (main effect, *p* = 0.156). The post-recovery increase in Treg to Tδγ ratio values was observed during the 2nd examination, but only in the supplemented group.

Statistical analysis showed that the values of Treg to CTL ratio were modulated by the exercise, but not by the supplementation (*p* < 0.001 and *p* = 0.771, respectively; Fig. 4d). Irrespective of the study group, no statistically significant changes in Treg to CTL ratio were documented during the 1st examination. During the 2nd examination, a significant post-recovery increase in Treg to CTL ratio values was observed in both supplemented group and placebo group.

Selected indices of complete blood count and TAC levels determined during the 1st and the 2nd examination are shown in Table 5. Irrespective of the examination term, the exercise-induced changes in WBC counts in athletes from both groups followed a similar pattern, with a post-exercise increase in this parameter and its return to the baseline level after a 24-h recovery. During the 1st examination, physical exercise did not exert a significant effect on the percentages of lymphocytes and granulocytes. During the 2nd examination, a significant post-recovery increase in granulocyte percentage was observed in LTE-supplemented athletes, along with a significant post-recovery decrease in lymphocyte percentages in both groups.

**Table 5:** Selected parameters of complete blood count and the level of total antioxidant capacity before and after supplementation with L-theanine (LTE) or placebo (Pla)

Parameters		Before supplementation			After supplementation			ANOVA		
		Pre-Exercise x ± SD	Post-Exercise x ± SD	Post-Recovery x ± SD	Pre-Exercise x ± SD	Post-Exercise x ± SD	Post-Recovery x ± SD	Exercise	LTE	Exercise x LTE
WBC [10^9^/L]	LTE	6.81 ± 1.47	11.16 ± 1.52*	6.53 ± 1.04†	6.25 ± 1.17	11.12 ± 1.96*	6.46 ± 1.51^†^	*< 0.0001*	*0.2236*	*0.7378*
	Pla	5.98 ± 1.27	10.1 ± 1.56*	5.75 ± 0.75^†^	6.03 ± 1.13	10.83 ± 1.91*	6.0 ± 0.74^†^			
Granulocytes [%]	LTE	46.0 ± 10.81	47.6 ± 3.85	52.6 ± 5.53	46.4 ± 3.69	41.9 ± 5.37	51.3 ± 6.25^†^	*< 0.0001*	*0.8803*	*0.4637*
	Pla	48.9 ± 5.68	47.8 ± 6.22	48.8 ± 5.44	45.5 ± 3.58	43.4 ± 7.35	49.9 ± 6.04			
Lymphocytes [%]	LTE	42.73 ± 7.85	44.59 ± 4.21	38.44 ± 6.04	43.48 ± 3.08	48.36 ± 4.93	38.79 ± 5.35^†^	*< 0.0001*	*0.5098*	*0.5920*
	Pla	43.19 ± 5.32	45.13 ± 6.59	42.83 ± 6.02	44.98 ± 3.39	47.62 ± 6.99	40.17 ± 6.19^†^			
TAC (mmol/l)	LTE	2.36 ± 0.38	1.78 ± 0.40*	2.11 ± 0.55	2.22 ± 0.44	1.80 ± 0.35	2.43 ± 0.41^†^	*< 0.0001*	*0.2035*	*0.5617*
	Pla	2.49 ± 0.22	1.65 ± 0.28*	2.18 ± 0.42	1.87 ± 0.46	1.69 ± 0.24	2.39 ± 0.46			

*WBC* White blood cells; *TAC* Total Antioxidant Capacity

*p < 0.05 – compared to Pre-Exercise value

^†^*p* < 0.05 – compared to Post-Exercise value

Statistical analysis demonstrated that TAC was modulated solely by physical exercise (*p* < 0.001). Before the supplementation (1st examination), a significant post-exercise decrease in serum TAC was observed in both study groups. During the 2nd examination, a post-recovery increase in TAC level was observed in athletes from the supplemented group, but not in those from the placebo group (Table 5).

None of the athletes reported adverse events throughout the period of L-theanine supplementation.

## Discussion

The present study, including members of Polish National Rowing Team, demonstrated that in the athletes supplemented with L-theanine, ergometer test contributed to a post-recovery decrease in IL-10 level (Fig. 1). The decrease in IL-10 level was associated with an increase in the values of IL-2 to IL-10 and IFN-γ to IL-10 ratios. The post-recovery values of both ratios in the supplemented group were significantly higher than at the baseline (Fig. 2). These changes might correspond to a favorable shift in Th1/Th2 balance toward Th1 caused by supplementation with theanine, which would be consistent with our research hypothesis. Published evidence suggests that supplementation with theanine may influence concentration of some cytokines. Li et al. [24] demonstrated that both IL-4 to IFN-γ ratio and serum concentrations of IL-10, IL-6 and IL-4 decreased with a growing dose of L-theanine administered to rats via a feeding tube.

Since IL-10 inhibits the activity of Th1 cells and NK and decreases the synthesis of Th1 cytokines (IL-2 and IFN-γ), its elevated concentrations may predispose to cellular immunosuppression [25–27]. In contrast, a decrease in IL-10 level boosts cellular immunity, which is vital not only in the context of URTI prevention but also for cancer control. One example of practical application of this knowledge is therapeutic suppression of IL-10 production with low-dose cyclophosphamide, which produces anti-metastatic effect due to a shift in Th1/Th2 balance toward Th1. In this context, previously published observations that IL-10 is a tumor growth factor and IFN-γ exerts a cytotoxic effect on metastatic cells seem to be particularly worth emphasizing [28]. Thus, the increase in IFN-γ to IL-10 ratio values observed after strenuous exercise in supplemented athletes but not in the controls seems to be particularly valuable finding.

However, the results of supplementation were different when the number of Tregs and some cytotoxic cells, NK, Tγδ and CTL, were considered (Fig. 3). During the 2nd examination (after supplementation), the post-recovery Treg counts in both placebo group and supplemented group were significantly higher than at the baseline (Fig. 3a). Moreover, the post-recovery CTL count in athletes from the supplemented group was significantly lower than immediately after the exercise (Fig. 3d). Finally, during the 2nd examination, the post-recovery values of both Treg to NK and Treg to Tγδ ratios in the supplemented group were significantly higher than immediately after the exercise (Fig. 4b and c). The interpretation of these findings is not straightforward. Theoretically, the supplementation might contribute to a decrease in the number of cytotoxic CTLs. Also, the post-recovery increase in the values of Treg to NK and Treg to TCR ratios may suggest that the supplementation with LTE promoted unfavorable changes in the proportion of cytotoxic lymphocytes to Tregs. However, these results should not be interpreted without consideration of the cytokines synthesized by those cells, since otherwise, any conclusions about beneficial or unfavorable effect of LTE on immunity might be biased. A decrease in CTL count should be linked to a decrease in the level of IL-10 synthesized by Th2 cells, since according to literature, IL-10 promotes growth and differentiation of CTLs [29]. Equally important is the observation that the post-exercise increase in CTL count observed during the 2nd examination, although not statistically significant, was more evident in the supplemented group than in the controls. However, after a 24-h recovery, mean CTL counts in both groups were essentially the same.

Our study did not demonstrate an effect of LTE supplementation on Tγδ count (Fig. 3c). It must be stressed, that the supplemented group and the placebo group did not differ significantly in terms of the concentrations of Th1 cytokines (IL-2 and IFN-γ) (Fig. 1a and d). However, the post-recovery number of CTLs (a source of Th1 cytokines) in the supplemented group was lower than immediately after the exercise (Fig. 3d). Thus, the decrease in IL-10 concentration and the increase in the values of IFN-γ to IL-10 and IL-2 to IL-10 ratios after supplementation with LTE might reflect higher activity of Tγδ cells in Th1 cytokine production. This is consistent with the results published by Mao et al. [30] who observed an inhibitory effect of IL-10 on the synthesis of Th1 cytokines by Tγδ cells, as well as with the findings reported by Bukowski et al. [10] according to whom, LTE administered at lower concentrations enhanced production of Th1 cytokines (IFN-γ, TNF-α) by Tγδ cells, and stimulated proliferation of those cells at higher concentrations.

In this study, we did not determine concentrations of other cytokines that theoretically could contribute to a shift in Th1/Th2 balance. Thus, we can only speculate that the post-supplementation decrease in CTL count and lack of changes in the number of other analyzed cytotoxic cells did not affect negatively cytotoxic potential of the athletes after the strenuous exercise. However, this hypothesis needs to be verified in further research.

Unlike Murakami et al. [11, 31], we did not observe statistically significant differences in the percentages of lymphocytes and granulocytes before and immediately after the ergometric test. Statistically significant changes, namely a decrease in the lymphocyte percentage in both groups and an increase in the percentage of granulocytes (the latter solely in the supplemented group) in relation to post-exercise values were observed no earlier than after the recovery (Table 5). In the study conducted by Murakami et al. [31], individuals from the control group showed a post-exercise increase in granulocyte percentage and a decrease in lymphocyte percentage, whereas a suppression of post-exercise neutrophilia and lymphopenia was observed in individuals supplemented with cystine and LTE. The discrepancies between our findings and the results published by Murakami et al. [31] might be associated with the use of different exercise loads. Nevertheless, the lack of post-exercise increase in granulocyte percentage and concomitant increase in lymphocyte percentage in the study conducted by those authors were most likely caused by cystine or its synergy with LTE. When administered separately, LTE caused an increase in granulocyte percentage, which was also observed in our present study (Table 5). According to Murakami et al. [31], the synergistic effect of cystine and LTE may contribute to an increase in glutathione concentration, which results in attenuation of oxidative stress, and thus, in lesser post-exercise neutrophilia and lymphopenia. It should be emphasized that also a training load specific for a given phase of the yearly cycle might exert an effect on the post-exercise changes in the study parameters (Table 2). In our present study, the strenuous ergometric test conducted before the supplementation (during the preparatory phase) did not induce any statistically significant changes in the percentages of granulocytes and lymphocytes. However, the change of training loads, namely an increase in the proportion of high-intensity exercises before the 2nd examination (competitive phase) was reflected by a post-recovery decrease in lymphocyte percentages, observed in both supplemented group and the controls (Table 5). It needs to be stressed that the competitive phase constitutes a serious strain for the athletes as despite a similar training volume (970 min and 960 min during the 1st and 2nd examination, respectively), the proportion of high and very high intensity exercises is markedly larger (Table 2). High exercise intensity is an important element of adaptation to the so-called “competitive” loads, but may also exert an unfavorable effect contributing to an immune impairment [32].

Moreover, our study demonstrated that supplementation with theanine contributed to a statistically significant post-recovery increase in TAC, which implies that after the exercise, the redox potential in athletes from the supplemented group was higher than in the controls. In turn, the increase in the percentage of granulocytes observed in the supplemented group at the end of the recovery period might result from a decrease in the concentration of anti-inflammatory IL-10 and resultant increase in the level of granulocyte-macrophage stimulating factor (GM-CSF) (not analyzed in this study), stimulating proliferation and activity of granulocytes [33–35].

## Conclusions

To the best of our knowledge, this was the first study to analyze the effect of L-theanine (LTE) on selected cytotoxic cells, Treg and some Th1 and Th2 cytokines in elite athletes exposed to strenuous exercise. After the 24-h recovery, athletes from the supplemented group presented with significantly lower CTL counts and significantly higher values of Treg to NK and Treg to CTL ratios than immediately after the exercise. Paradoxically, however, the post-recovery concentrations of IL-10 in LTE-supplemented athletes were significantly lower and the values of IFN-γ to IL-10 and IL-2 to IL-10 ratios significantly higher than at the baseline. This implies that despite a decrease in CTL and unfavorable increase in Treg ratio to some cytotoxic cells, some elements of cellular immunity might be enhanced due to inhibition of IL-10 synthesis and a beneficial shift toward production of Th1 cytokines. Thus, it can be stipulated that despite the decrease in the number of some cytotoxic cells (CTLs) and an increase in the proportion of Tregs to CTLs observed after the supplementation, LTE may exert a beneficial effect on a disrupted Th1/Th2 balance, as shown by the decrease in IL-10 concentration. The decrease in IL-10 level, occurring regardless of higher TAC, might be a reason behind the post-recover increase in granulocyte percentage in the supplemented group.

## Abbreviations

CTL: Cytotoxic T lymphocyte
IL: Interleukin
INT-γ: Interferon gamma
LA: Lactic acid
NK: Natural killer
OTS: Overtraining syndrome
RDA: Recommended dietary allowance
TAC: Total antioxidant capacity
Th: Human T-helper
Treg: Regulatory T-cell
Tδγ: T*-*cell gamma delta
URTA: Upper respiratory tract infections
WBC: White blood cells
